# Evaluation of the Short-Term Effects of Antimicrobial Stewardship in the Intensive Care Unit at a Tertiary Hospital in China

**DOI:** 10.1371/journal.pone.0101447

**Published:** 2014-07-07

**Authors:** Dapeng Hou, Qiushi Wang, Cuihua Jiang, Cui Tian, Huaqing Li, Bo Ji

**Affiliations:** 1 Department of Intensive Care Unit, the Affiliated Hospital of Taishan Medical College, Taian, Shandong, China; 2 Department of Thoracic Surgery, the Affiliated Hospital of Taishan Medical College, Taian, Shandong, China; National Center for Biotechnology Information (NCBI), United States of America

## Abstract

Antibiotic abuse can lead to antibiotic resistance, which is a severe problem in China. The purpose of this study is to evaluate the short-term effects of antimicrobial stewardship strategies, including formulary restriction, preauthorization, perioperative quinolone restriction, and control of total antibiotic consumption in the ICU at a tertiary hospital in China. After implementation of antimicrobial stewardship, the total antibiotic consumption in the ICU significantly decreased. The defined daily doses (DDDs) per 100 patient-days decreased from 197.65 to 143.41; however, the consumption of cephalosporins increased from 53.65 to 63.17 DDDs. Significant improvements in resistance to amikacin, gentamicin, ciprofloxacin, ofloxacin, ceftriaxone, ceftazidime, and piperacillin in Enterobacteriaceae and resistance to ceftazidime, imipenem, and meropenem in non-fermenting Gram-negative rods were observed. In addition, the initial use of no antibiotics or of a single antibiotic significantly increased (P<0.001) and the use of two antibiotics in combination significantly decreased (P<0.001). Our results demonstrate that implementation of antimicrobial stewardship in a short period in the ICU dramatically reduced antibiotic consumption and significantly improved antibiotic resistance, which leads to more reasonable antibiotic selections by ICU physicians.

## Introduction

Since antibiotics were first applied in clinical settings, more varieties have emerged, resulting in the recovery of numerous critically infected patients [1]. However, excessive antibiotic consumption has also caused increases in pharmaceutical fees, adverse drug events, and antibiotic resistance [2]. This problem is particularly serious in China because of the country's antibiotic prescription practices, strong incentives for overprescribing, and over-the-counter availability of antibiotics [3]. Comparison of the patterns of antibiotic resistance among bacteria of clinical relevance in China, Kuwait and the USA demonstrated that China had the highest level of antibiotic resistance and the most rapid growth rate of resistance among the three countries [4]. Such antibiotic overuse and the rapid growth rate of antibiotic resistance are particularly serious in intensive care units (ICUs) because patients in ICUs usually have serious health problems. An investigation demonstrated that in China's ICUs, the antibiotic resistance rate of common pathogens is increasing every year [5].

The Chinese government is aware of the harm caused by antibiotic overuse and has developed national policies to regulate its use. From April to August in 2011, according to the policy of the Ministry of Health of China and the Bureau of Health of Shandong Province, all public hospitals in Shandong province began to follow strict antimicrobial stewardship strategies. It has been reported that antimicrobial stewardship is an efficient way to improve antibiotic resistance and to reduce patient cost by optimizing antimicrobial prescriptions [6]. Numerous studies have evaluated the long-term and short-term impacts of antimicrobial stewardship based on antibiotic consumption, antibiotic resistance, and clinical outcomes. In general, reasonable antimicrobial stewardship can significantly reduce the total consumption of antibiotics and improve antibiotic resistance, which can lead to better clinical outcomes [6]–[9]. China still lacks systematic studies on the short-term and long-term effects of antimicrobial stewardship. Evaluation of the effects of antimicrobial stewardship may provide useful information to further improve antimicrobial stewardship strategies. This study describes the implementation of new antimicrobial stewardship practices in our hospital and evaluates the short-term effects of antimicrobial stewardship strategies as implemented in the ICU.

## Materials and Methods

### Research ethics

This study was conducted with the permission and supervision of the Ethics Committee of the Affiliated Hospital of Taishan Medical College, Taishan, Shandong Province, China. Oral consent was obtained from patients and documented in our research notebook for use of their clinical records in this study. Written consent was not obtained because of illiteracy problem of many patients. The ethics committee mentioned above approved the oral consent protocol. All patient records/information was anonymized prior to analysis.

### Antimicrobial stewardship strategies

**Figure 1:**
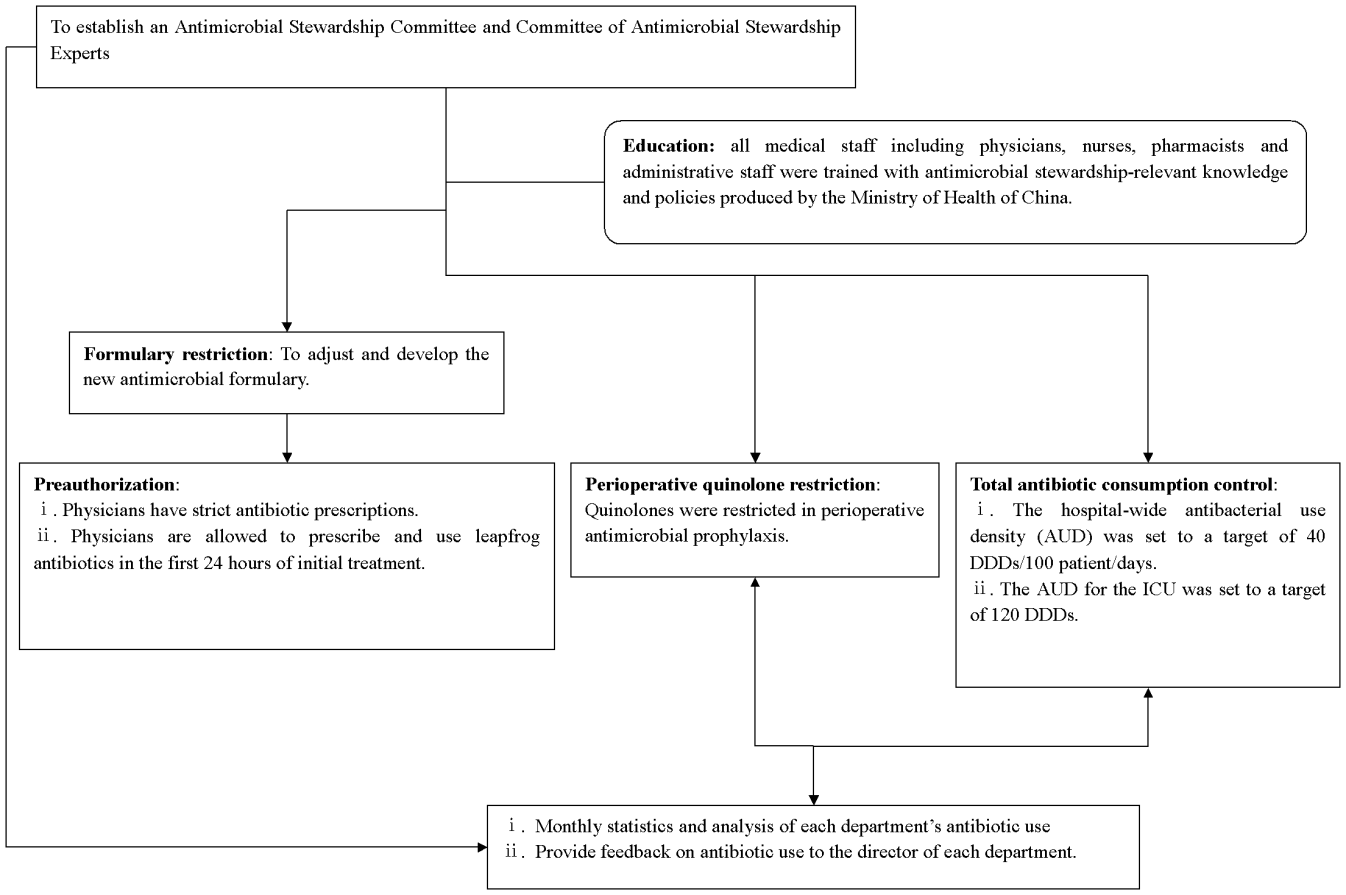
Flow chart of antimicrobial stewardship strategies applied in the ICU at a tertiary hospital in China. These strategies primarily included formulary restriction, preauthorization, perioperative quinolone restriction, and control of total antibiotic consumption.

The Affiliated Hospital of Taishan Medical College is a tertiary teaching hospital with 700 beds. The ICU of our hospital is comprehensive with 12 beds and annually handles 550 patients. From April to August in 2011, antimicrobial stewardship strategies were put into practice, including formulary restrictions, preauthorization requirements, perioperative quinolone restriction and control of total antibiotic consumption. The specific strategies and classification standards are described in the flow-chart in Figure 1 and in Appendixes S1 and S2. We compared the data before and after implementation of the antimicrobial stewardship (the ‘before’ period was October 2010 to March 2011, and the ‘after’ period was October 2011 to March 2012).

### Data collection

To evaluate the effects of antimicrobial stewardship in antibiotic consumption, data on antibiotic resistance, clinical outcomes, demographic and clinical data of patients, antibacterial use density (AUD), antibiotic resistance level, and initial antibiotic selection were collected and compared before and after the implementation of antimicrobial stewardship.

Demographic and clinical data included age, gender, APACHE II score, primary (infectious or non-infectious) disease when admitted to the ICU, the application of antibiotics within the 30 days prior to the transfer, the application of glucocorticoid therapy within the 30 days prior to the transfer, whether the patient accepted the treatment of mechanical ventilation in the ICU, days of treatment with mechanical ventilation, length of ICU stay, death, and whether the death was correlated with infection. Previous application of glucocorticoid therapy referred to the application of glucocorticoids, oral or intravenous, within 30 days, excluding aerosol inhalation.

Antibiotic consumption was calculated based on the WHO standards [10], which is the cumulative antibacterial use density (AUD) expressed as DDDs per 100 patient-days. The number of DDDs per 100 patient days is often used as an indicator for the selection pressure exerted by antibiotics in the hospital setting. The results of microbial cultivation were summarized by WHONET. Isolated pathogenic strains were grouped into three categories: Enterobacteriaceae, non-fermenting Gram-negative rods, and Gram-positive cocci. All the isolates were identified by standard microbiological methods, and susceptibility testing was performed by the disk diffusion test according to the recommendations of the Clinical and Laboratory Standards Institute [11] and with Vitek II AST-No. 17 GNB cards (bioMerieux, Marcy l'Etoile, France). Duplicate isolates collected from the same patient within the same hospital stay were counted only once.

Data on antibiotic use before admission to the ICU were collected, including intravenous and oral antibiotic application 30 days before admission to the ICU, use, antibiotic varieties and how many varieties used. Statistics on the initial antibiotic use after admission to the ICU were also collected, including whether used, the antibiotic varieties used, and whether monotherapy or combination therapy was used.

### Statistical analysis

Data were analyzed using SPSS 11.0. All tests were two-sided, and significance was defined as a P-value≤0.05. Categorical variables are presented as a percentage and were compared using χ^2^ analysis. Continuous variables are presented as the mean ± standard deviation and were compared using one-way ANOVA.

## Results

### General information

There was no significant difference in sex ratio, age, APACHE II score, primary disease, proportion of infectious diseases, and prior glucocorticoid use between, before, and after the implementation of antimicrobial stewardship. No significant differences in ICU length of stay, the number of days on mechanical ventilation, mortality, or infection-related mortality was observed between these two groups. However, we found a higher proportion of no antibiotic therapy or single antibiotic therapy in the ‘after’ period, which may be explained by the hospital-wide implementation of antimicrobial stewardship strategies (Table 1).

**Table 1:** Comparison of demographic and clinical data of patients before and after antimicrobial stewardship.

Characteristic	Before (n = 259)	After (n = 279)	P
Male/female	170/89	187/92	0.734
Age	53.10±19.43	54.59±18.07	0.357
APACHE II score	21.46±8.04	21.04±9.14	0.578
Primary diseases			0.998
Medical diseases	84	90	
Surgical diseases	80	86	
Trauma	95	103	
Infectious diseases/non-infectious	50/209	44/235	0.281
Transfer from Infectious department/General department	106/153	139/140	0.038
Antibiotic use before			0.011
Non	178(68.7%)	214(76.7%)	
Single	33(12.7%)	39(14.0%)	
Two	36(13.9%)	19(6.8%)	
Three	11(4.2%)	4(1.4%)	
More than three	1(0.4%)	3(1.1%)	
Glucocorticoid use before	19/240	19/260	0.812
With or without ventilator application	140/119	177/102	0.027
Ventilator days	3.38±3.54	3.30±5.29	0.867
ICU days (median)	2	2	0.148
All-cause death/survival	40/219	32/247	0.176
Death associated with infection/others	11/248	8/271	0.386

### Significant decrease of antibiotic consumption after the implementation of antimicrobial stewardship

Our studies demonstrated a significant decrease in the hospital-wide total antimicrobial consumption (from 69.69 DDDs to 50.76 DDDs, a decrease of 27.16%). Concomitantly, the ICU total antibiotic consumption also decreased significantly after implementation of the antimicrobial stewardship strategies. The DDDs per 100 patient-days decreased from 197.65 to 143.41, which is a decrease of 27.44%. The consumption of quinolones, imidazoles, other β-lactams, and macrolides decreased significantly. Particularly, the DDDs of quinolone per 100 patient-days decreased sharply from 49.82 to 7.74. The DDDs of glycopeptide and carbapenem did not decrease but increased slightly. Accompanied by the increase in the consumption of cephalosporins, the DDDs per 100 patient-days increased from 53.65 to 63.17, which is an increase of 17.74% (Table 2).

**Table 2:** Changes of antimicrobial consumption (DDDs/100 patient/days) after the antimicrobial stewardship.

Antibiotics	ICU			Hospital-wide		
	Before	After	Change (%)	Before	After	Change (%)
Penicillins	2.72	0.27	−90.07	3.37	1.58	−53.12
Cephalosporins	53.65	63.17	+17.74	23.95	30.09	+25.64
Carbapenems	16.44	17.05	+3.71	0.48	0.45	−6.25
Other *Β*-lactams	10.11	0.59	−94.16	12.29	3.75	−69.49
Aminoglycosides	0.47	0.00	__	0.15	0.06	−60.00
Tetracyclines	28.63	29.76	+3.95	0.85	0.81	−4.71
Macrolides	11.43	3.39	−70.34	5.01	3.58	−28.54
Glycopeptides	3.63	3.73	+2.75	0.11	0.09	−18.18
Quinolones	49.82	7.74	−84.46	14.32	3.40	−76.26
Imidazoles	14.63	10.61	−27.48	8.13	6.08	−25.22
Antifungal Agents	6.01	6.96	+15.81	0.43	0.34	−20.94
Others	0.11	0.14	+27.27	0.60	0.53	−11.67
Total	197.65	143.41	−27.44	69.69	50.76	−27.16

### Dramatic improvement of antibiotic resistance after the implementation of antimicrobial stewardship

In the ICU, antibiotic resistance for Gram-negative bacilli improved significantly after the implementation of antimicrobial stewardship strategies. The percentage of Enterobacteriaceae isolates with resistance to amikacin, gentamicin, ciprofloxacin, ofloxacin, ceftriaxone, ceftazidime and piperacillin noticeably decreased. In addition, the percentage of isolate resistance to ceftazidime, imipenem and meropenem also decreased for non-fermenting Gram-negative rods. The percentage of isolates with resistant Gram-positive cocci did not change significantly (Table 3).

**Table 3:** Changes of percentage of bacterial isolates with antibiotic resistance (%) after antimicrobial stewardship.

Antibiotic	Enterobacteriaceae			Non-fermenting Gram-negative rods			Gram-positive cocci		
	Before	After	P	Before	After	P	Before	After	P
Amikacin	82.6	44.7	0.004	88.6	81.4	0.356			
Gentamycin	91.3	39.5	<0.001				90.9	100	1.000
Ciprofloxacin	91.3	44.7	<0.001	91.4	84.7	0.525	90.9	88.9	1.000
Ofloxacin	91.3	42.1	<0.001						
Ceftriaxone	95.7	50.0	<0.001						
Ceftazidime	78.3	39.5	0.003	85.7	64.4	0.026			
Cefepime	52.2	36.8	0.241	60.0	52.5	0.482			
Imipenem	4.3	0	0.377	85.7	59.3	0.007			
Piperacillin	87.0	57.9	0.018	80.0	72.9	0.438			
Ampicillin/Sulbactam	56.5	42.1	0.275						
Tobramycin				94.3	79.7	0.054			
Levofloxacin				88.6	81.4	0.356			
Meropenem				88.6	62.7	0.007			
Minocycline				11.4	23.7	0.143			
Piperacillin/Tazobactam				77.1	66.1	0.258			
Oxacillin							100	100	___
Tetracycline							100	100	___
Vancomycin							0	0	___

### Selection of initial antibiotic use after the implementation of antimicrobial stewardship

The initial antibiotic selection was also influenced by the antimicrobial stewardship. After implementation of antimicrobial stewardship, the initial selection of no antibiotic or single antibiotic increased significantly (5.0% vs 2.3%, 78.9% vs 20.5%), and the initial selection of two antibiotics were significantly lower (16.1% vs 74.1%). In the ‘before’ period, between the infectious and the non-infectious group, there was no significant difference in initial selection concerning whether to use and how many varieties used (χ^2^ = 3.339, P = 0.297), whereas in the ‘after’ period, the initial selection between the two groups was significantly different (χ^2^ = 8.487, P = 0.012), with a higher proportion of no antibiotic and single antibiotic use (5.5% vs 2.3% and 81.3% vs 65.9%, respectively) and a lower proportion of two antibiotic use (13.2% vs 31.8%) in the non-infectious group (Table 4).

**Table 4:** Comparison of the ICU physicians' initial antibiotic selection before and after antimicrobial stewardship.

Combination therapy	Before No.(%) of patients (n = 259)	After No.(%) of patients (n = 279)	Before^1^ No.(%) of patients		After^2^ No.(%) of patients	
			Infectious diseases (n = 50)	Non-Infectious (n = 209)	Infectious diseases (n = 44)	Non-Infectious (n = 235)
Unused	6(2.3%)	14(5.0%)	0(0%)	6(2.9%)	1(2.3%)	13(5.5%)
Single antibiotic	53(20.5%)	220(78.9%)	8(16.0%)	45(21.5%)	29(65.9%)	191(81.3%)
Two antibiotics	192(74.1%)	45(16.1%)	39(78.0%)	153(73.2%)	14(31.8%)	31(13.2%)
Three antibiotics	8(3.1%)	0(0%)	3(6.0%)	5(2.4%)	0(0%)	0(0%)

Initial antibiotic combination therapy in ‘before’ and ‘after’ period: χ^2^ = 217.924, P<0.001.

1 Before antimicrobial stewardship, χ^2^ = 3.339, P = 0.297.

2 After antimicrobial stewardship, χ^2^ = 8.487, P = 0.012.

## Discussion

The extensive application of antibiotics in the ICU has received increasing attention. A survey in Germany, with 40 ICUs included, indicated that patients in the ICU consumed a considerable amount of antibiotics with an average consumption of 1351 DDDs/per 1,000 patient days [12]. The use of antibiotics cures many patients with serious infections and allows them to recover; however, it has also promoted the emergence and spread of multidrug-resistant bacteria [13]. Many ICU physicians and infection control experts have made attempts, including formulary restriction, preauthorization, antimicrobial cycling, de-escalation of therapy, and combination therapy to control drug resistance [7], [14]–[16]; however, these studies have produced inconsistent results [9]. In this study, we found that both the hospital-wide AUD and the AUD of the ICU significantly decreased. The antibiotic resistance of Gram-negative bacillus, especially Enterobacteriaceae, reduced significantly after the implementation of antimicrobial stewardship. In addition, we also observed more reasonable initial antibiotic selections by ICU physicians. Our study further supports the nation-wide implementation of antimicrobial stewardship in China.

For antibiotic consumption in the ICU, the total DDDs dropped significantly from 197.65 to 143.4, which is a decrease of 27.44%. Of the total antibiotic consumption, quinolones, other β-lactams, and imidazoles decreased significantly, and the ratio of the initial use of antibiotics also declined. The consumption of a number of alternative antibiotics, including cephalosporins and non-controlled antibiotics increased because their prescriptions were loosely controlled; however, our results indicated that cephalosporin resistance did not increase, despite the increase of cephalosporin consumption. In addition, Enterobacteriaceae exhibited a reduction in resistance to ceftriaxone and ceftazidime and non-fermenting Gram-negative rods exhibited a reduction in resistance to ceftazidime.

We presume that the decreased consumption of antibiotics and improvement in antibiotic resistance achieved from the antimicrobial stewardship were mainly attributed to the control of the total antibiotic consumption and to regional control. Although the targeted DDDs for the ICU (120 DDDs) were higher than for other departments, the Antimicrobial Stewardship Committee gave a monthly statistical analysis report to the director of the ICU, who was required to improve antibiotic control with the goal of progressively lowered use, especially when the AUD was higher. Such administrative pressure would ideally be transmitted to each ICU physician. ICU physicians would then have to implement the antimicrobial stewardship strategies strictly, so that the increase of alternative antibiotic consumption and related rise in resistance would not occur. In addition, at the same time, hospital-wide, regional and even national implementations of antimicrobial stewardship strategies are much more likely to have been the key to the positive results achieved. The majority of the ICU patients were transferred from other departments or other hospitals. Patients transferred from so-called infection departments that have heavy antibiotic use and high resistance rates accounted for 45.54% of individuals with infectious disease patients accounting for 37%. These patients might already have resistant strains before they are transferred to the ICU; therefore, if antimicrobial stewardship strategies are only implemented in the ICU, and not included in other departments, the effect of the implementation in the ICU would be seriously affected.

In this study, there was a significant difference (P = 0.011) in the ‘before’ and ‘after’ periods of antibiotic use before admission to the ICU (Table 1). There was a higher proportion of no antibiotic and single antibiotic therapy before admission to the ICU in the ‘after’ period, indicating its synchronization with the hospital-wide decrease of antibacterial use. There were also relatively small decreases of antibiotic use density in the subgroup of critical patients who were most likely to be transferred to the ICU. As these patients had a lower probability of being exposed to a variety of antibiotics or strong antibiotic density before admission to the ICU, the decline of past exposure may also be helpful in the decline of antibiotic resistance in the ICU.

We noted that compared with the ‘before’ period, the consumption of carbapenems did not decline; instead, an increase was seen in their use. Interestingly, there was a decline in the resistance to imipenem and meropenem for non-fermenting Gram-negative rods. This phenomenon may be associated with strict quinolone prescription regulations and the rigid decrease of quinolone use. Many studies have demonstrated that excessive quinolone use is an independent risk factor of *Pseudomonas aeruginosa* resistance to imipenem. By limiting the use of quinolone antibiotics, *P. aeruginosa* resistance to imipenem and meropenem can be reduced in addition to decreasing the second-generation carbapenem antibiotic selection pressure [17], [18]. Preclinical studies have revealed that there may be a common pathway in the resistance mechanism of *P. aeruginosa* to quinolones and carbapenems, such as a loss or decrease in levels of the outer membrane porin protein, OprD, or the overexpression of the MexAB-OprM efflux system. Such mechanisms may explain why an improvement of quinolone resistance in *P. aeruginosa* was accompanied by an improvement of carbapenem resistance [19]–[21].

We noted that, in addition to the decrease of antibacterial use density and the improvement of resistance, the implementation of antimicrobial stewardship also had impacts on the antibiotic selections of ICU physicians [8]. In regards to the initial antibiotic selection after admission to the ICU, there was a significant difference between the ‘before’ and ‘after’ periods. Combinations of two antibiotics were more common in the ‘before’ period, whereas single antibiotic therapy was more common in the ‘after’ period. Implementation of antimicrobial stewardship strategies, including education and use restriction, led the ICU physicians to change their prescription habits, actively or passively, and made them more inclined to initially choose single antibiotic therapy. However, the change of the antibiotic selection tendency had no impact on the total mortality of ICU patients. On the other hand, before implementation of the antimicrobial stewardship strategies, the infectious status had little impact on the ICU physicians' selection of single, combination or no antibiotic therapy (Table 4). That is, in regards to antibiotic selection, ICU physicians may not use patient infectious status as the main consideration, which results in excessive antibiotic application. Such physician behavior was altered after the intervention and patient infectious status has been one of the critical factors for ICU physicians to consider antibiotic selection (Table 4). An initial antibiotic combination therapy for patients with infectious diseases took over a relatively high proportion of the prescribed antibiotics, indicating more reasonable antibiotic therapy practices of ICU physicians after the intervention. In summary, antimicrobial stewardship led to reasonable antibiotic selections in the ICU, which can directly contribute to the reduction of antibiotic use and may also decrease rates of antibiotic resistance.

There are some limitations to this study. This was a passive intervention study with only a single-center and a small sample size. Encouragingly, 33 tertiary hospitals, including ours, have formed a collaborative network for monitoring ICU antimicrobial resistance. The effect of the implementation of antimicrobial stewardship strategies is now being analyzed under the assessment of this collaboration. In addition, the present study only investigated the short-term effects of antimicrobial stewardship but the long-term effects should also be considered in future studies.

In summary, implementation of antimicrobial stewardship strategies, including formulary restriction, preauthorization, restriction of perioperative antimicrobial prophylaxis, and control of total antibiotic consumption, significantly reduced antibiotic use in a relatively short period. The resistance of Gram-negative bacteria to some antibiotics was improved to some extent. Simultaneously, the selection of antibiotics in the ICU became more reasonable. Abuse of antibiotics and resulting antibiotic resistance has become a major clinical and public health problem in China. Our results further support the national implementation of antimicrobial stewardship strategies; however, the long-term effects of these strategies require continuous tracking, monitoring and assessment.

## Supporting Information

Appendix S1 Antimicrobial Stewardship.
(DOCX)

Appendix S2 Antimicrobial Classification.
(DOCX)
