# Supplementary material for: Evaluation of the Short-Term Effects of Antimicrobial Stewardship in the Intensive Care Unit at a Tertiary Hospital in China
Source: PLoS One. 2014 Jul 7;9(7):e101447. doi: 10.1371/journal.pone.0101447 (PMC4084822; doi:10.1371/journal.pone.0101447)
Supplement: Appendix S1 — Antimicrobial Stewardship. (DOCX) [file pone.0101447.s001.docx]

**Appendix S1:** Antimicrobial stewardship.

| Antimicrobial Stewardship: |
| --- |
| 1. Antimicrobials were divided into three classes: non-restricted, restricted, and controlled. 2. Residents could only prescribe non-restricted antibiotics and attending doctors could prescribe non-restricted and restricted antibiotics. Only associate chief physicians and chief physicians could prescribe controlled antibiotics with the consent of the Infection Control Committee experts. However, physicians were allowed to prescribe and use leapfrog antibiotics in the first 24 hours of the initial treatment. 3. Quinolones were restricted in perioperative antimicrobial prophylaxis 4. The hospital’s goal for DDDs was less than 40/100 patient-days. 5. Antimicrobial stewardship was associated with medical quality assessment. Hospital administrators were responsible for negotiation with directors of DDD excessive departments. |
