# Supplementary material for: Evaluation of the Short-Term Effects of Antimicrobial Stewardship in the Intensive Care Unit at a Tertiary Hospital in China
Source: PLoS One. 2014 Jul 7;9(7):e101447. doi: 10.1371/journal.pone.0101447 (PMC4084822; doi:10.1371/journal.pone.0101447)
Supplement: Appendix S2 — Antimicrobial Classification. (DOCX) [file pone.0101447.s002.docx]

**Appendix S2:** Antimicrobial classification.

| The three antimicrobial classifications were as follows: |
| --- |
| 1. Non-restricted antimicrobials: penicillin, piperacillin, amoxicillin, cefazolin, cefradine, cefuroxime, ceftriaxone, SMZ, doxycycline, tetracycline, erythromycin, azithromycin (per os), clindamycin, lincomycin, gentamycin, amikacin, ciprofloxacin, ofloxacin, levofloxacin, metronidazole, tinidazole, nitrofurantoin, nystatin, fluconazole (per os), and itraconazole (per os) 2. Restricted antimicrobials: mezlocillin, ampicillin+sulbactam, piperacillin+tazobactam, cefprozil, cefotaxime, cefoperazone, sulbactam+cefoperazone, ceftazidime, cefminox, azithromycin (parenteral), tobramycin, moxifloxacin, fluconazole (parenteral), and voriconazole (parenteral) 3. Controlled antimicrobials: tigecycline, cefepime, cefpirome, aztreonam, imipenem, meropenem, lomefloxacin, fleroxacin, vancomycin, norvancomycin, teicoplanin, linezolid, amphotericin B, voriconazole (parenteral), itraconazole (parenteral), caspofungin, and micafungin |
